# Evaluation of Weed Species for Host Status to the Root-Knot Nematodes *Meloidogyne enterolobii* and *M. incognita* Race 4

**DOI:** 10.2478/jofnem-2024-0017

**Published:** 2024-04-22

**Authors:** Tanner Schwarz, Chitra, Katherine Jennings, Adrienne Gorny

**Affiliations:** Department of Entomology and Plant Pathology, North Carolina State University, Raleigh, NC 27695; Department of Horticultural Science, North Carolina State University, Raleigh, NC 27695

**Keywords:** greenhouse bioassay, guava root-knot nematode, southern root-knot nematode, root galling, reproductive factor

## Abstract

Weeds that compete with valuable crops can also host plant-parasitic nematodes, acting as a source of nematode inoculum in a field and further damaging crops. The host status of 10 weed species commonly found in North Carolina, USA, was determined for the root-knot nematodes *Meloidogyne enterolobii* and *M. incognita* race 4 in the greenhouse. Each weed species was challenged with 5,000 eggs/plant of either *M. enterolobii* or *M. incognita* race 4, with five replicate plants per treatment in two separate greenhouse trials. Root galling severity and total number of nematode eggs per root system were recorded 60 days after inoculation. Reproduction factor (Rf = final nematode population/initial nematode population) was calculated to determine the host status of each weed species to *M. enterolobii* and *M. incognita* race 4. Four weed species (*Datura stramonium, Digitaria sanguinalis, Senna obtusifolia,* and *Cyperus esculentus*) were poor hosts (Rf < 1) to both nematode species, and roots of these weed plants did not display galling. Four weed species (*Ipomoea hederacea, Amaranthus palmeri, Portulaca pilosa,* and *Ipomoea lacunosa*) were hosts (Rf > 1) to both nematode species, and all had observable root gall formation. *Sida rhombifolia* and *Cyperus rotundus* were poor hosts to *M. enterolobii* but susceptible hosts to *M. incognita*. This study documents a differential host status of some common weeds to *M. enterolobii* and *M. incognita* race 4, and these results highlight the necessity of managing root-knot nematodes through controlling weeds in order to protect valuable crops.

Weeds are undesired plants that compete with valuable crops for water, nutrients, sunlight, and space (Chauhan, 2020). Crops with sunlight or nutrient deficiencies caused by the presence of weeds are more prone to reduced yields and are more susceptible to infection by pathogens and disease (Reberg-Horton et al., 2011; Dentika et al., 2021). Weeds can also harbor plant pathogens and act as alternate hosts for pathogens, including plant-parasitic nematodes, which can reproduce and increase in population, making effective field management more difficult (Rich et al., 2008; Dentika et al., 2021; Lopez et al., 2021). Therefore, weeds are a global issue that requires proper management to prevent crop loss (Vila et al., 2021); a 100% yield loss can result if weeds are left uncontrolled (Chauhan, 2020). It is estimated that weeds cause greater crop loss than either pathogens or insects (Fried et al., 2017) and their effect, combined with nematodes in the field, may be more detrimental to crop production (Dentika et al., 2012).

The global crop loss due to plant-parasitic nematodes is estimated at $157 billion annually (Singh et al., 2015; Abdel-Sattar et al., 2020). Previous research has shown that weeds can host many pathogens, including plant-parasitic nematodes. It has been reported that species of *Meloidogyne*, *Heterodera, Pratylenchus*, *Helicotylenchus*, and numerous other nematodes can infect and multiply on weeds (Barlow, 2011; Ntidi et al., 2012; Lopez et al., 2021; Rocha et al., 2021). Nematodes can survive on weeds in the presence or absence of a field cash crop, thus providing a source of inoculum for the following crops and increasing disease risk (Kaur et al., 2007; Rich et al., 2008). Plant-parasitic nematodes are more difficult to control to a manageable threshold at high population numbers (Castagnone-Sereno, 2012), and weeds allow nematodes to multiply, applying greater disease pressure on valuable crops. Methods such as plant genotypic resistance, crop rotation, soil tillage, organic amendments, soil solarization, chemicals, and biocontrols have been documented to reduce nematode populations to varying success (Zasada et al., 2010); however, these tactics may be negligible if plant-parasitic nematode populations are surviving and increasing on weeds. In addition, although nematicides are an effective tool for managing nematodes (Oka, 2020), certain weeds have been documented to provide protection for nematodes from nematicides and adverse environmental conditions (Thomas et al., 2004). The presence of weeds as alternative hosts may offset control tactics. Therefore, weed management is vital for effective nematode management.

It is important to understand which weeds are host to plant-parasitic nematodes in order to improve management. *Meloidogyne enterolobii* Yang and Eisenback, 1983, (syn. *M. mayaguensis*) is a highly virulent root-knot nematode (RKN) species that causes significant damage on a broad range of plants (Castagnone-Sereno, 2012). Considered one of the most damaging RKN species (Ye et al., 2021), *M. enterolobii* is a relatively recently emergent RKN species in the United States, and there is still limited knowledge about its interactions with weeds. *Meloidogyne incognita* race 4, the southern RKN, is widely distributed throughout the world and is one of the most common species of RKN in the United States, including in North Carolina (Schwarz et al., 2020). Like other RKNs, *M. incognita* has a broad host range that includes numerous important global crops. Weeds may be providing a secondary host refuge for these nematode species, causing further difficulties in nematode management and an increase in crop loss. Therefore, the objective of this study was to determine the host status of 10 common weed species in North Carolina to *M. enterolobii* and *M. incognita* race 4 to inform nematode management recommendations.

## Materials and Methods

### Planting, inoculation, and evaluation of weed species for their host status to *Meloidogyne enterolobii* and *M. incognita*

Ten species of weeds were evaluated for their host status (susceptibility or resistance) to the RKNs *Meloidogyne enterolobii* and *M. incognita* race 4. The weed species included: Palmer amaranth (*Amaranthus palmeri*), yellow nutsedge (*Cyperus esculentus*), purple nutsedge (*Cyperus rotundus*), jimsonweed (*Datura stramonium*), large crabgrass (*Digitaria sanguinalis*), entireleaf morningglory (*Ipomoea hederacea*), pitted morningglory (*Ipomoea lacunosa*), pink purslane (*Portulaca pilosa*), sicklepod (*Senna obtusifolia*), and prickly sida (*Sida rhombifolia*). Seeds of each weed species were provided by Dr. Katie Jennings of the Fruit and Vegetable Weed Science program at NC State University (Raleigh, NC) and are representative of eastern North Carolina weed populations. Seeds were planted in plastic cone containers with dimensions 25.4 cm deep by 3.8 cm wide at the top, containing a 1:1 steam sterilized sand to soil mixture in the greenhouse. Three weeks after planting, each plant was inoculated with either 5,000 eggs of *M. enterolobii* or 5,000 eggs of *M. incognita* by pipetting the inoculum solution into a 3 cm deep depression at the base of the stem. For each trial, five plants of each weed species were inoculated separately with *M. incognita* and *M. enterolobii*, with each plant considered as a replicate. Plants were blocked by nematode species, and weed species were randomized within the block. Plants were maintained in the greenhouse at 25°C to 28°C with no supplemental lighting. The *M. enterolobii* and *M. incognita* race 4 isolates (confirmed using species specific PCR; Schwarz et al., 2020) used in each trial were extracted from 2-month-old cultures maintained on “Rutgers” tomato plants in the greenhouse using the NaOCl extraction method of Hussey and Barker (1973), quantified under an inverted compound light microscope at 40× magnification, and inoculated onto the weed plants the same day.

The host status of the weed plants was evaluated 60 days postinoculation. Each plant was destructively harvested and cut at the crown to isolate the roots to assess for root galling and total nematode eggs in each root system. Roots were gently removed from the pots and rinsed free of soil with cool tap water. Visual root gall severity ratings were assigned based on the total percentage of the root system galled, following the schematic of Bridge and Page (1980). RKN eggs were then extracted from each root system following the NaOCl method described by Hussey and Barker (1973), recovered on a 25-µm mesh sieve, and collected in a final volume of 50 mL with water. The total number of nematode eggs extracted from each root system was determined by counting three, 1 mL aliquot of the egg extraction solution at 40× magnification using an inverted compound microscope (Nikon TM, Nikon Instruments, Melville, NY) and averaging the three counts. Reproduction factor (Rf = final total egg count/initial egg inoculum) was calculated and used to determine the host status of each weed. An Rf value less than or equal to 0.01 (Rf ≤ 0.01) was considered a non-host. An Rf value greater than 0.01 and less than 1.0 (0.01 > Rf < 1.0) was considered a poor host. An Rf value greater than or equal to 1 but less than 5 (1.0 ≤ Rf < 5.0) was considered a host plant, and an Rf value greater than 5 (Rf > 5) was considered a good host plant. Host and good host plants were considered susceptible, while non-host and poor-host plants were considered resistant.

### Data analysis

Data from each trial were evaluated separately due to a significant effect of the trial, and all statistical analyses were conducted within RStudio (v. 4.2.1; R Core Team, 2022). The assumptions of normality and homogeneity of variances were evaluated using the Shapio-Wilks (function “shapio.test”) and Levene’s test (function “leveneTest” within the “car” package), respectively. The tests indicated significant deviation from a normal distribution, so a log_10_(x+1) transformation was applied to the Rf and eggs per gram of root, which rectified the assumption. All following statistical analysis were performed using the log_10_(x+1) transformed data; however non-transformed means of replicates from each trial are presented in the results to aid in interpretation. To assess for statistical differences between weed species in Rf and eggs per gram of root, treatments were evaluated using analysis of variance with replicate as a random effect. Where significant differences were observed, Fisher’s least significant difference test was used to separate means (function “LSD.test” within the “agricolae” package).

## Results

### Weed species response to *Meloidogyne enterolobii*

**Figure 1:**
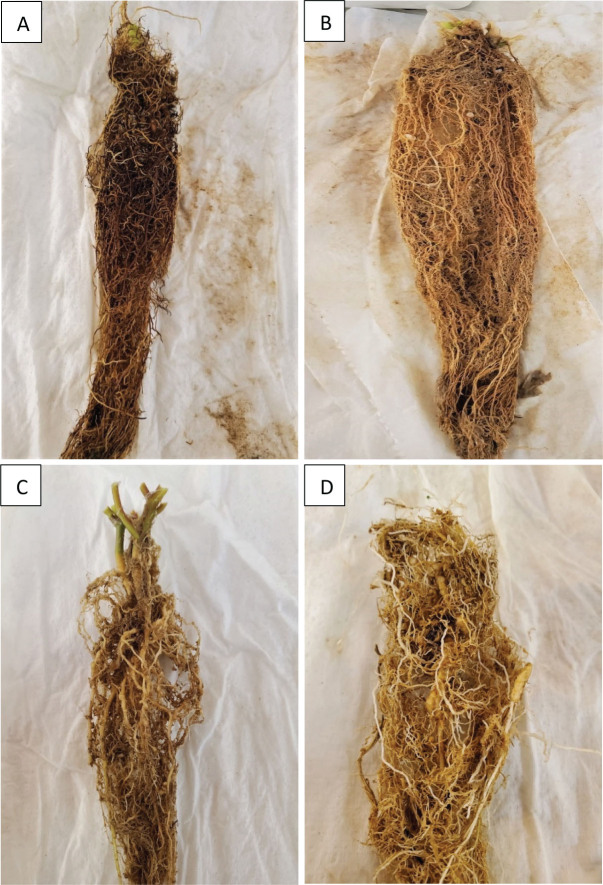
(A) Sicklepod (*Senna obtusifolia*) resistant to *Meloidogyne incognita* race 4. (B) Resistant large crabgrass (*Digitaria sanguinalis*) inoculated with *M. enterolobii* showing no nematode gall formation. (C) Pink purslane (*Portulaca pilosa*) infected with *M. incognita* race 4; susceptible host displaying galling damage. (D) Entireleaf morningglory (*Ipomoea hederacea*) susceptible to *M. enterolobii* with galling symptoms.

Among the 10 weed species tested, there were both susceptible (host and good host) and resistant (poor host) weeds to *Meloidogyne enterolobii*. Jimsonweed, large crabgrass, prickly sida, purple nutsedge, sicklepod, and yellow nutsedge were all considered poor hosts to *M. enterolobii*, as each had an Rf value of less than 1.0 but greater than 0.01 (Table 1) and were significantly different (*P* < 0.001) from Rf values of weed species evaluated as susceptible hosts. Among these poor hosts, purple nutsedge had the highest Rf value at 0.53 and 0.44 in Trials 1 and 2, respectively (Table 1). Pricky sida had the lowest Rf value among the poor hosts at 0.07 and 0.05 in Trials 1 and 2 (Table 1). Gall ratings supported the poor host status; the root systems for all replicates determined as poor hosts contained zero observable galls, scored as 0% (Table 1). Palmer amaranth was considered susceptible and a host plant (1.0 ≤ Rf < 5.0), and entireleaf morningglory, pink purslane, and pitted morningglory were determined to be susceptible and good hosts, as each had an Rf value greater than 5.0 (Table 1). Pink purslane had the highest Rf value (9.64) among susceptible hosts in Trial 1. The lowest Rf value among susceptible host weeds was 2.69 on Palmer amaranth, which still represents a significant increase to the initial inoculum. All weeds considered hosts contained obserable root galling on all replicates (Figure 1). The highest gall rating was 50% on pink purslane; the lowest gall rating score determined among susceptible plants was 5% on Palmer amaranth. The host status determined for each weed species in Trial 1 was consistent with the host status determined in Trial 2. There were no weeds considered as non-hosts (Rf ≤ 0.01) to *M. enterolobii*.

**Table 1:** Host status of 10 weed species common in North Carolina, USA to the RKN *Meloidogyne enterolobii* in two separate greenhouse trials. Weed plants were inoculated with 5,000 eggs of *M. enterolobii* and evaluated for egg production and root galling 60 days post inoculation. Values are the mean of five replicate plants per trial. Values followed by the same letter within the column are not significantly different at the α = 0.05 level.

**Weed Species**	**Rf^a^**		**Gall Rating^b^**		**R/S**
	
	**Trial 1**	**Trial 2**	**Trial 1**	**Trial 2**	
Palmer amaranth (*Amaranthus palmeri*)	2.69b	3.35b	5b	5b	S
Yellow nutsedge (*Cyperus esculentus*)	0.13a	0.09a	0a	0a	R
Purple nutsedge (*Cyperus rotundus*)	0.53a	0.44a	0a	0a	R
Jimsonweed (*Datura stramonium*)	0.08a	0.06a	0a	0a	R
Large crabgrass (*Digitaria sanguinalis*)	0.06a	0.07a	0a	0a	R
Entireleaf morningglory (*Ipomoea hederacea*)	5.36b	5.14b	14b	20b	S
Pitted morningglory (*Ipomoea lacunosa*)	8.26b	8.03b	19b	10b	S
Pink purslane (*Portulaca pilosa*)	9.64b	7.18b	50b	25b	S
Sicklepod (*Senna obtusifolia*)	0.15a	0.10a	0a	0a	R
Prickly sida (*Sida rhombifolia*)	0.07a	0.05a	0a	0a	R

a Reproduction factor, Rf= final total egg count/initial inoculum egg count. Rf values less than 1 are considered resistant (R) and a poor host; those greater than 1 are considered susceptible (S) and a host or good host. No non-host weed species were observed in this study.

b Root gall severity rating (the visual estimate of the percent of the root system affected by galls, following the schematic of Bridge and Page, 1980).

### Weed species response to *Meloidogyne incognita* race 4

Among the 10 weed species tested, there were both susceptible (host and good host) and resistant (poor host) weeds to *M. incognita* race 4. Jimsonweed, large crabgrass, sicklepod, and yellow nutsedge were considered poor hosts, as each had an Rf value between 0.01 and 1.0 (Table 2) and were significantly different (*P* < 0.001) from Rf values of the susceptible host plants. Yellow nutsedge had the largest Rf value among poor host weeds to *M. incognita*, with an Rf of 0.24 in both Trials 1 and 2. Jimsonweed had the lowest Rf value among the poor host weeds at 0.06 and 0.04 in Trials 1 and 2, respectively. No root galls were observed on any of the root systems identified as a poor host (Figure 1). Entireleaf morningglory, Palmer amaranth, and prickly sida were considered susceptible and hosts (1.0 ≤ Rf < 5.0). Pink purslane, pitted morningglory, and purple nutsedge were considered susceptible and good hosts, as each had a significant Rf value greater than 5.0 (Table 2). Pitted morningglory had the highest Rf value among susceptible weeds at 14.23 in Trial 1 (Table 2). Prickly sida had the lowest Rf value among susceptible weeds at 1.80 and 2.04 in Trials 1 and 2, respectively (Table 2). Root galling severity ratings supported the designations of a susceptible host status, as all replicates contained observable root galling. Pink purslane had the highest root galling severity score at 60% and 35% in Trials 1 and 2, respectively (Table 2), and entireleaf morningglory, prickly sida, and purple nutsedge had the smallest gall rating scored among susceptible host weeds to *M. incognita* at 5% (Table 2). No weed was considered as a non-host (Rf ≤ 0.01) to *M. incognita* race 4.

**Table 2:** Host status of 10 weed species common in North Carolina, USA to the RKN *Meloidogyne incognita* race 4 in two separate greenhouse trials. Weed plants were inoculated with 5,000 eggs of *M. incognita* and evaluated for egg production and root galling 60 days post inoculation. Values are the mean of five replicate plants per trial. Values followed by the same letter within the column are not significantly different at the α = 0.05 level.

**Weed Species**	**Rf^a^**		**Gall Rating^b^**		**R/S**
	
	**Trial 1**	**Trial 2**	**Trial 1**	**Trial 2**	
Palmer amaranth (*Amaranthus palmeri*)	2.84c	3.48c	9b	5b	S
Yellow nutsedge (*Cyperus esculentus*)	0.24a	0.24a	0a	0a	R
Purple nutsedge (*Cyperus rotundus*)	12.87c	4.36c	5a	5b	S
Jimsonweed (*Datura stramonium*)	0.06a	0.04a	0a	0a	R
Large crabgrass (*Digitaria sanguinalis*)	0.08a	0.05a	0a	0a	R
Entireleaf morningglory (*Ipomoea hederacea*)	4.14c	4.40c	5a	10b	S
Pitted morningglory (*Ipomoea lacunosa*)	14.23c	9.84c	10b	10b	S
Pink purslane (*Portulaca pilosa*)	8.79c	11.31c	60b	35c	S
Sicklepod (*Senna obtusifolia*)	0.08a	0.07a	0a	0a	R
Prickly sida (*Sida rhombifolia*)	1.80b	2.04b	5a	5b	S

a Reproduction factor, Rf= final total egg count/initial inoculum egg count. Rf values less than 1 are considered resistant (R) and a poor host; those greater than 1 are considered susceptible (S) and a host or good host. No non-host weed species were observed in this study.

b Root gall severity rating (the visual estimate of the percent of the root system affected by galls, following the schematic of Bridge and Page, 1980).

## Discussion

Weeds are detrimental to agriculture production. For example, annual crop loss due to weeds in North America is estimated at $16 billion for soybeans and $27 billion for corn (Soltani et al., 2016, 2017). Weeds may also impact agricultural production through their ability to host plant pathogens such as nematodes. The results obtained in this research indicate that several weeds are host to *M. enterolobii* and *M. incognita* race 4 and thus can increase populations of these nematode species in the soil, and may act as green bridges or pathogen reservoirs for present and future crops. Once RKNs establish in a field, it is difficult to eradicate them or reduce their populations to non-threatening thresholds (Lambert and Bekal, 2002). By providing a refuge for nematodes, weeds can be devastating for effective nematode management, and through the results observed in this study, may annul, to some degree, the effect of management tactics such as chemical or cultural control.

Two weed species, prickly sida and purple nutsedge, had differential host status, depending on the nematode species. Prickly sida was considered a poor host to *M. enterolobii* (Rf values of 0.07 and 0.05; Table 1), but susceptible to *M. incognita* (Rf values of 1.80 and 2.04 Table 2). Although the Rf values for *M. incognita* on prickly sida are not considerably large, the values are still greater than 1 and therefore indicate some nematode reproduction occurred. Purple nutsedge was also identified as resistant and a poor host to *M. enterolobii* (Rf values of 0.53 and 0.44; Table 1), but susceptible to *M. incognita* (Rf values of 12.87 and 4.36; Table 2). At a practical level, this differential host response highlights the importance of identifying both the nematode species and weed species present in a field management context, so that the relative risk to the cash crop can be evaluated.

The other eight weed species in this study were considered either poor or susceptible hosts to both species of RKN. Both entireleaf and pitted morningglory were host to both RKN species tested here. Pitted morningglory had previously been reported as host to *M. arenaria* (Rodriguez-Kebana et al., 1977; Rich et al., 2008), but this study represents the first evaluation of the weed’s host status to *M. incognita* and *M. enterolobii*. Morningglory weeds are botanically related to cultivated sweetpotato (*Ipomoea batatas*), and thus may have similar host profiles to common RKNs. For example, Clark and Watson (1983) demonstrated that *M. incognita* was pathogenic to all morningglory species and sweetpotato cultivars tested. Here, jimsonweed was found to be a non-host to both *M. enterolobii* and *M. incognita* race 4. Extracts of jimsonweed had an inhibitory effect on egg hatching and J2 activity (Oduor-Owino, 1993; Babaali et al., 2017), which supports further inquiry into how this weed resists infection by the nematode. Although purposeful cultivation of this weed for nematode management is not recommended, this weed may serve as a promising source for a biocontrol agent. In the present study, pink purslane was found to be host to both nematode species. To our knowledge, no other studies have evaluated its host status directly, yet previous work has documented that the related species common purslane (*Portulaca oleracea*) and showy purslane (*P. grandiflora*) are host to *M. incognita* (Mani and Hinai, 1996; Brito et al., 2008; Rich et al., 2008), indicating that this plant genus in general may be host to *M. incognita*.

Results of the present study corroborate previous research regarding the host status of certain weeds to *M. enterolobii* and *M. incognita*. Similar to the present study, Kokalis-Burelle and Rosskopf (2012) found that yellow nutsedge is a poor host to *M. incognita.* Prickly sida was found to be susceptible to *M. incognita* (Davis and Webster, 2005; Belle et al., 2017). Kaspary et al. (2022) determined Palmer amaranth to be susceptible to *M. incognita*. As in the present study, Kaur et al. (2007) concluded that large crabgrass was resistant to *M*. *incognita* race 4 and *M. enterolobii* (published using the synonym *M. mayaguensis*) and sicklepod was a poor host for *M. incognita*.

However, some discrepancies between the present study and previous works were also identified. Kaur et al. (2007) found sicklepod to be a good host for *M. enterolobii*, whereas our research determined sicklepod was a poor host. Belle et al. (2017) found purple nutsedge to be resistant to *M. incognita*, and Mendes et al. (2020) concluded that both yellow and purple nutsedge are susceptible to *M. enterolobii* and *M. incognita*, while the present work determined purple nutsedge to be susceptible to *M. incognita* but resistant to *M. enterolobii* (Table 1).

Differences in the host status of weeds between previous studies and the present study may be due to the genetic variability of weeds (Sterling et al., 2004; Mangolin et al., 2012). With potential for high genetic variability and short life cycles, it is likely that the seeds from weed populations used in the studies differed genetically, and this could impact the ability of the nematode to infect the plant. In addition, distinct nematode populations may show differential host preferences, depending on their origin and selection in the field, with physiological races of *Meloidogyne spp.* being distinguished by their ability to infect certain plants (Barker et al., 1985). These differences in physiological races, along with diverse weed populations, may have contributed to the variable host status results among studies (Rich et al., 2008). Several previous research studies either did not indicate which races of *M. incognita* were used, or used races other than race 4. For example, Schroeder et al. (1993) found that yellow and purple nutsedge were susceptible to *M. incognita* race 3. Tedford and Fortnum (1988) also used a race 3 isolate of *M. incognita* in an extensive host status assay of weeds in South Carolina. Kaur et al. (2007) used *M. incognita* race 4 in their studies; only sicklepod overlapped between their study and the present study, yet both studies concluded that sicklepod was a poor host to race 4. The present study used *M. incognita* race 4, and *M. incognita* race 3 is known to differ from *M. incognita* race 4 in its ability to parasitize tobacco in the North Carolina Differential Host Test (Taylor and Sasser, 1978). With this known host range, variability observed in well-studied cash crops, and variability in other, less-studied plants is also plausible and may have contributed to the discrepancies between studies. Furthermore, Rutter et al. (2021) found that two *M. enterolobii* populations isolated from North and South Carolina had differential responses when inoculated to identical sweetpotato genotypes.

Although certain weeds were considered resistant and poor hosts to either *M. enterolobii* or *M. incognita* race 4 in the present study, the weeds still supported a low level of nematode reproduction, as eggs were extracted and quantified from the root system. No weed was determined as a non-host for RKN, herein defined as displaying an Rf ≤ 0.01. Due to this finding, it is the authors’ recommendation that producers manage all weeds regardless of the weed or nematode species present in the field, as a small proportion of the RKN population may be maintained on these poor host weeds over time. To further illustrate the importance of weed management in the field, Barlow (2011) and Thomas et al. (2004) stated that yellow and purple nutsedge weeds (*Cyperus* spp.) have a symbiotic relationship with RKNs. These nutsedge weeds increased production of tubers (the overwintering stage of the nutsedge plant) when infected by RKNs. The nematodes appear to be able to use these tubers to survive during unfavorable environmental conditions or during a period that lacks a primary host plant. An increase in nutsedge tuber size and multiplication of RKN populations increases the potential for further crop loss, potentially due to nutsedge pressure, nematode pressure, or both. Furthermore, Thomas et al. (2004) found that yellow and purple nutsedge tubers protected RKN from nematicides, allowing a greater proportion of the nematode individuals to survive the nematicide application and increase in population.

The present study reports the host status of 10 weeds common in North Carolina, USA and identified differential host status among several of these weed species. The study also highlights the importance of weed management in the field in order to also manage plant-parasitic nematodes and protect valuable crops. This research contributes to the knowledge of weed–RKN interactions and pathogen management.
